# Skin Protection by Carotenoid Pigments

**DOI:** 10.3390/ijms25031431

**Published:** 2024-01-24

**Authors:** Jolanta Flieger, Magdalena Raszewska-Famielec, Elżbieta Radzikowska-Büchner, Wojciech Flieger

**Affiliations:** 1 Department of Analytical Chemistry, Medical University of Lublin, Chodźki 4A, 20-093 Lublin, Poland; 2 Faculty of Physical Education and Health, University of Physicl Education, Akademicka 2, 21-500 Biała Podlaska, Poland; raszewska.famielec@gmail.com; 3 Department of Plastic, Reconstructive and Maxillary Surgery, National Medical Institute of the Ministry of the Interior and Administration, Wołoska 137 Street, 02-507 Warszawa, Poland; elzbieta.radzikowska@gmail.com; 4 Chair and Department of Anatomy, Medical University of Lublin, K. Jaczewskiego 4, 20-090 Lublin, Poland; wwoj24@wp.pl

**Keywords:** photosynthetic pigments, carotenoids, UV protection, skin photoprotection, skin photodamage, nanoformulations

## Abstract

Sunlight, despite its benefits, can pose a threat to the skin, which is a natural protective barrier. Phototoxicity caused by overexposure, especially to ultraviolet radiation (UVR), results in burns, accelerates photoaging, and causes skin cancer formation. Natural substances of plant origin, i.e., polyphenols, flavonoids, and photosynthetic pigments, can protect the skin against the effects of radiation, acting not only as photoprotectors like natural filters but as antioxidant and anti-inflammatory remedies, alleviating the effects of photodamage to the skin. Plant-based formulations are gaining popularity as an attractive alternative to synthetic filters. Over the past 20 years, a large number of studies have been published to assess the photoprotective effects of natural plant products, primarily through their antioxidant, antimutagenic, and anti-immunosuppressive activities. This review selects the most important data on skin photodamage and photoprotective efficacy of selected plant carotenoid representatives from in vivo studies on animal models and humans, as well as in vitro experiments performed on fibroblast and keratinocyte cell lines. Recent research on carotenoids associated with lipid nanoparticles, nanoemulsions, liposomes, and micelles is reviewed. The focus was on collecting those nanomaterials that serve to improve the bioavailability and stability of carotenoids as natural antioxidants with photoprotective activity.

## 1. Introduction

The spectrum of solar radiation that reaches the Earth includes wavelengths ranging from 290 to 3000 nm [1]. Electromagnetic radiation produces different biological effects in humans depending on the frequency or, equivalently, the wavelength. Low-energy radiation, infrared, radio waves, and microwave fields are non-ionizing radiation (NIR). The short, high-frequency waves, i.e., gamma radiation (10^20^–10^24^ Hz, <10^−12^ m) and X-radiation (10^17^–10^20^ Hz, 1 nm^−1^ pm), are hazardous to living beings. Due to the high photon energy, this range of radiation can cause ionization by breaking atomic bonds. Humans are constantly exposed to low levels of ionizing radiation. This is known as natural background radiation. It comes mainly from sunlight, cosmic rays, naturally radioactive materials on Earth, or the anthropogenic environment.

**Figure 1:**
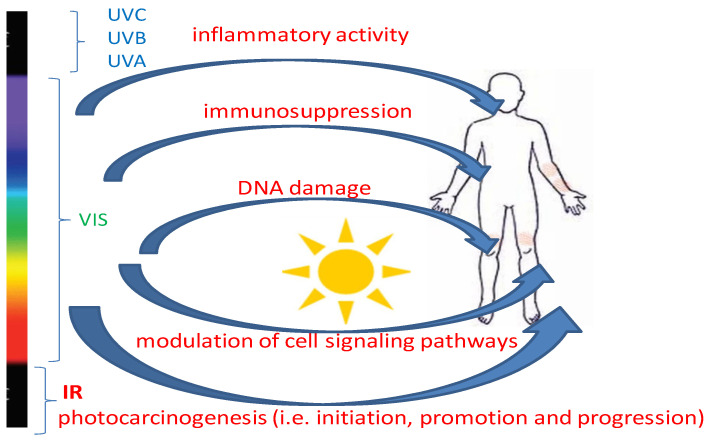
Factors contributing to skin photodamage.

The effect of exposure to intense optical radiation (ultraviolet > visible light > infrared) is to excite electrons in the tissue, resulting in heating or burns. Due to the low penetrability of optical radiation, the effects mainly affect surface tissues, i.e., the retina and skin [2,3]. While infrared radiation energy can be used in therapeutics due to its beneficial effects, especially on connective tissue reconstruction and vitamin D synthesis (eustress) by activating 7-dehydrocholesterol, the photochemical effects of ultraviolet (400 nm–1 nm, 10^15^–10^17^ Hz, UVR) are detrimental. Excessive, long-term exposure to UVR can cause eye diseases (cataracts, retinal degeneration), skin dysfunctions in the form of burns, photoaging, skin cancer, and even systemic effects in the form of a weakened immune system. It is not only UVR that is dangerous. It turns out that free radicals are formed in the skin after exposure to a wide range of wavelengths, from UVB (280 nm) through VIS (700 nm) to near-infrared (NIR), both in vivo and ex vivo [4,5]. Visible light has been shown to be responsible for about half of the total oxidative load on the skin [6]. High doses of infrared radiation also cause the formation of reactive singlet oxygen and hydroxyl radicals in human skin [7]. Photodamage to the skin results primarily from the occurrence of chronic inflammation, immunosuppression, and photocarcinogenesis (Figure 1).

**Figure 2:**
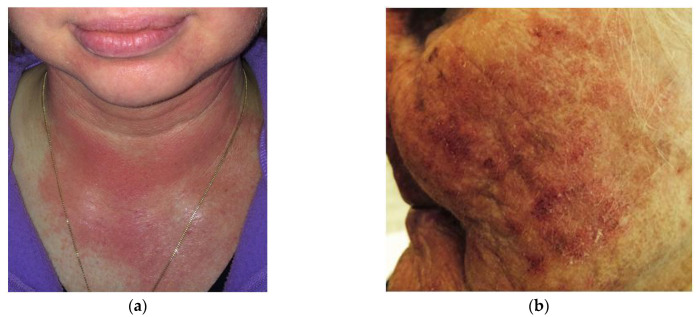

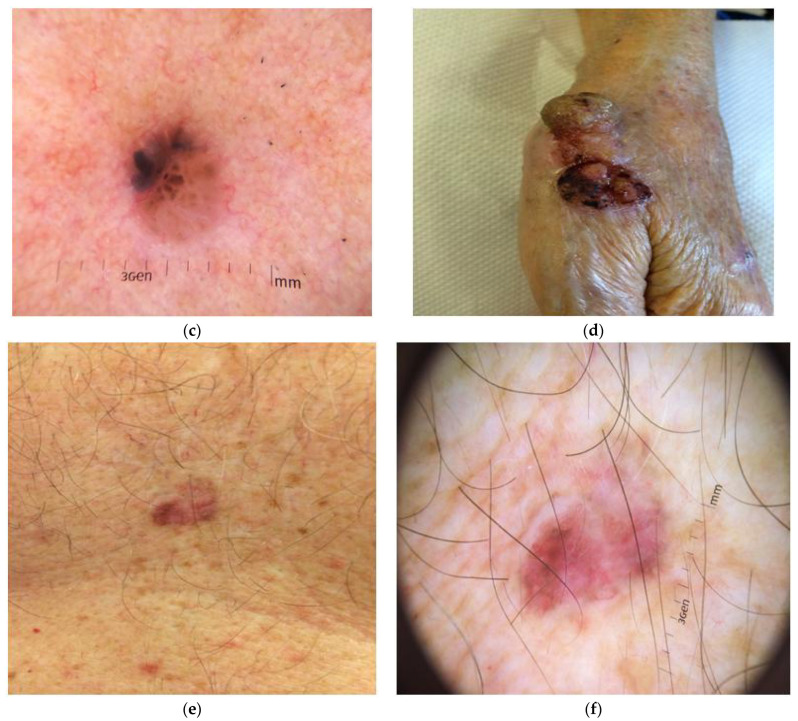
Examples of delayed-type hypersensitivity cutaneous reactions to a photoactivated allergen applied to the skin, long-term cutaneous effects of chronic exposure to UV—photoaging and photocancerogenesis—and skin tumors induced by reduced immune reactivity following chronic exposure to UV: (**a**) photoallergic contact dermatitis (PCD); (**b**) multiple actinic keratoses located on the checks; (**c**) dermoscopy—basal cell carcinoma (BCC); (**d**) squamous cell carcinoma (SCC); (**e**,**f**) dermoscopy—melanoma.

Photodamage of the skin may affect the epidermis and dermis, including hyperkeratosis, keratinocyte dysplasia, and skin elastosis. The clinical picture of photoaging skin with actinic keratosis is classified as a precancerous lesion that may lead to the development of squamous cell carcinoma (SCC) (Figure 2).

A method of mitigating the risk of UVR-induced damage is the use of endogenous and exogenous sunscreens and oral or topical antioxidants. Human skin, as a protective barrier, has at its disposal many enzymatic antioxidants, i.e., superoxide dismutase and catalase, and non-enzymatic ones [8], which are heterogeneously distributed in the stratum corneum (SC). It can be seen that the SC is dominated by non-enzymatic, exogenous antioxidants, which enter the SC from the blood during keratinization. Antioxidants also reach the skin surface with the secretion of sweat and/or sebum [9,10,11]. Photoprotection, both topical and systemic, uses different mechanisms of action, i.e., antioxidant, anti-inflammatory, antimutagenic, and anti-immunosuppressive functions, blocking photocarcinogenesis. Micronutrients, vitamins, and natural secondary metabolites of plant origin, i.e., polyphenols and carotenoids, have photoprotective potential [12]. Both topical and systemic photoprotection have advantages and limitations. Topical photoprotection in the form of UVR-absorbing sunscreens has a short half-life on the skin, poor systemic effects, and possible side effects [13,14]. In contrast, oral photoprotective agents attenuate oxidative stress, inflammation, and induce immunosuppression but do not provide a direct barrier against high-energy photons [12,15].

There are many studies in the literature on the support of skin protection against photodamage with plant-based products. Plant pigments used in photosynthesis are provided with chromophoric groups responsible for intense absorption (ε ~ l05 M^−1^ cm^−1^) over almost the entire range of the solar spectrum (330–900 and 1000–1100 nm) [15]. The apparent gap covering the 900–1000 nm range is understandable given that there is no competing system capable of absorbing light in the water absorption range at λ = 960 nm [16]. Chlorophylls (a, b, d) and the bacteriochlorophylls (BChl) have absorption in the range 330–480 nm and 630–1050 nm, respectively. The so-called “green gap” is filled by the absorption of chlorophyll C, the carotenoids, and the biliproteins. Carotenoids are useful pigments for photoprotection due to the high density of vibrational states and the very low energy of carotenoid triplets, which is usually below that of singlet oxygen (1274 nm, 7849 cm1, or 93.9 kJ/mol). In plants, carotenoids, together with chlorophyll, are not only involved in the use of light for photosynthesis but also play an important role in photoprotection by protecting plant tissues from oxidative stress [17].

This review summarizes achievements regarding the use of selected plant photosynthetic pigments from the carotenoid group to protect the skin against UVR radiation.

Particular attention was paid to the experimental models used and the mechanisms of photoprotective action. Previous achievements in the production of stable and bioavailable nanoformulations (lipid nanoparticles, nanoemulsions, liposomes, and micelles) containing carotenoids as natural sun protection agents were also summarized.

## 2. Photoprotective Function of the Skin/Skin Phototypes

The skin’s susceptibility to sunburn as well as its ability to tan, the so-called skin phenotype, was first classified in 1975 by Fitzpatrick (FSP) [18,19]. According to this traditional FSP classification, which remains the gold standard, skin type I, in Caucasians, is the most at-risk and susceptible to melanoma and non-melanoma skin cancers. Criticism of the FST classification relates to its application in ethnically diverse populations, e.g., people of Japanese origin, Indian origin, African origin, and Hispanic origin [20,21,22,23]. In recent years, new objective classification systems have been developed, e.g., the skin cancer phototype classification (SCP), the human eumelanin skin color scale, or the objective measure of constitutive pigmentation developed by Del Bino and Bernard based on the individual typology angle (ITA) [24]. Recently, new tools based on artificial intelligence have become available as smartphone applications [25]. Different classifications of skin phenotypes are summarized in Table 1.

**Figure 3:**
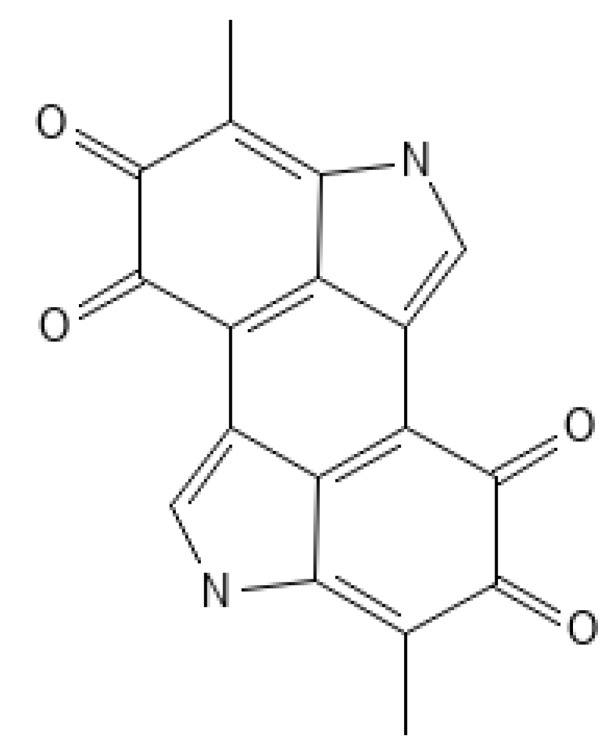
Chemical structure of melanin.

**Figure 4:**
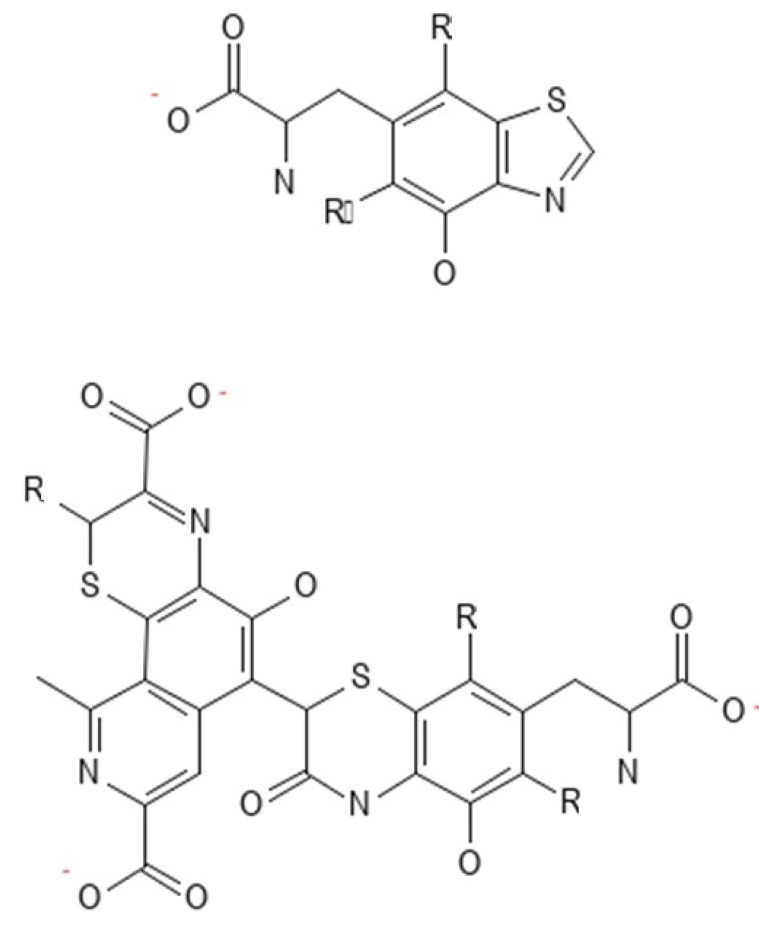
Chemical structure of pheomelanin.

**Figure 5:**
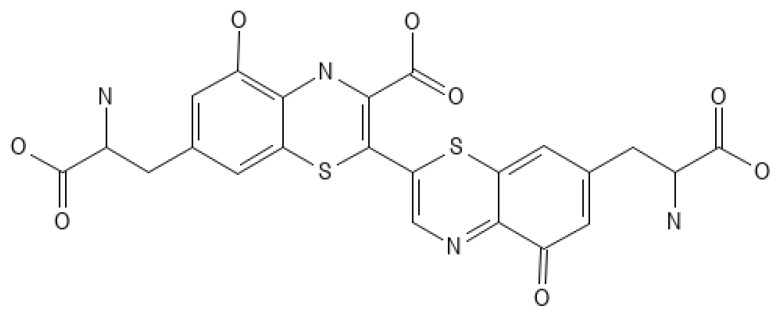
Chemical structure of eumelanin.

The protective function in the skin is performed by melanin (Figure 3), an insoluble polymer composed of antioxidant monomers, namely 5,6-dihydroxyindole (DHI) and 5,6-dihydroxyindole carboxylic acid (DHICA), which is able to absorb UVR, thus reducing skin damage. Melanin is produced by melanocytes located in the epidermis. Through melanosomes, it is transported to the keratocytes. The higher content of melanosomes in dark-skinned skin makes these individuals less likely to develop skin cancer compared to light-skinned individuals [47,48]. UVR causes the oxidation of melanin and its spatial redistribution; the delayed tanning (DT) effect as a result of new melanin synthesis raises the sun protection factor (SPF) twofold, which provides a photoprotective effect, protecting DNA from further UV damage [1,49]. In addition to melanin, there are other pigments in human skin, such as brown-black eumelanin and an alkali-soluble red-yellow pheomelanin (Figure 4), which contain benzothiazine monomers. Dark skin contains 3–6 times more melanin and eumelanin (Figure 5) and larger melanosomes compared to white skin. Therefore, dark skin allows 7.4 percent of UVB and 17.5 percent of UVA rays to pass through the epidermis, while white skin allows 24 percent of UVB and 55 percent of UVA rays to pass through [50].

The photoprotective effect of eumelanin was utilized to synthesize new pigments by the oxidative polymerization of DHICA [51]. DHICA-melanins show significant absorption in the UVA region [52]. The problem encountered in the full use of DHICA-melanins was their poor solubility and susceptibility to (photo)degradation. This research team developed a method for obtaining MeDHICA-melanin by oxidative polymerization of the DHICA methyl ester [53,54]. The obtained polymer counteracted UVA-induced oxidative stress in a study on human keratinocyte (HaCaT) cell lines.

## 3. The Effect of Radiation (R) on Skin Damage

Due to the presence of the ozone layer, only part of the light reaches the earth. The ozone layer absorbs almost 100% of UVC, transmitting a fraction of UVB (0.1%), UVA (5%), and IR (39–45%) [55]. The effect of radiation has different photobiological impacts on the skin, and it can be harmful as well as beneficial depending on the combination of wavelength, dose, and radiation intensity [56]. The biological effect of radiation will always be proportional to the total dose of energy absorbed, which is the product of irradiation and exposure time.

Generally, longer wavelengths with lower energy penetrate skin more deeply than shortened ones; thus, for instance, UVA rays are more penetrating than UVB ones. In the case of erythema (sunburn), increasing the wavelength reduces the radiation energy and, at the same time, reduces the effectiveness of the erythema. Therefore, UVB is most effective in causing erythema compared to UVA, which is a thousand times less effective. However, in the case of IR, the biological effect depends more on the radiation intensity than the total absorbed dose. Moreover, tissues with higher fat content are less susceptible to IR because fat conducts heat less well compared to other tissues [56]. While the mechanisms involved in UVR-induced skin damage have been thoroughly explored, this relatively new area of research covers the effects of visible light (VL) on the skin. Table 2 summarizes the general effects related to exposure to the broad radiation spectrum.

### 3.1. Ultraviolet Radiation (UVR)

Ultraviolet radiation (UVR, 280–400 nm) is divided into three categories: short-wave UVC (100–280 nm), medium-wave UVB (280–315 nm), and long-wave UVA (315–400 nm). This division of UVR radiation was proposed in 1932 by the Second International Congress on Light [64]. There are slightly different ranges of UVR radiation in photodermatology, namely UVC (200–290 nm), UVB (290–320 nm), and UVA (320–400 nm) [65]. While UVC radiation does not reach the Earth because it is scattered and absorbed by the ozone layer, UVB and UVA radiation reach approximately 10% and over 90%, respectively, contributing to various skin pathologies [2,66].

Over the last decades, it has been confirmed that UVR radiation initiates the process of carcinogenesis in the skin [67], responsible for the development of non-melanoma skin cancers (NMSC) and melanoma [47,68].

There is a continuous increase in the incidence of skin cancers such as basal cell carcinoma (BCC), squamous cell carcinoma (SCC), and melanoma, which constitute 40% of all human cancers [69,70]. The carcinogenic spectrum of UVR is in the range of 280–320 nm. UVB radiation is absorbed by keratinocytes in the epidermis, which contributes to the formation of the so-called erythema resulting from sunburn [71]. UVA radiation with wavelengths shorter than 320 nm penetrates deeper into the dermis. People using tanning beds exposed to high doses of UVA are particularly vulnerable to skin cancer [72], just like Caucasians [47]. It turns out that in Caucasian people, five times more UVR penetrates the epidermis than through the epidermis of black people [73]. The minimum erythemal dose (MED) of UVB is 1000 times lower than that of UVA in Caucasian skin. In addition to skin complexion, UVR penetration also depends on many other factors, such as the thickness of the corneal layer of the epidermis, skin hydration, age, gender, medications taken, radiation dose (time of day, latitude, reflection of the environmental surface, e.g., sand versus snow, temperatures) [1]. People with reduced immunity and previous episodes of severe sunburn in childhood are also considered risk factors for actinic keratosis and carcinogenesis [74].

The process of photocarcinogenesis is initiated by the interaction of radiation with nucleic acids, leading to oxidative DNA damage and immunosuppression (suppression of Langerhans cells in the epidermis and T lymphocytes in the peripheral blood) [75]. The effects of radiation are mainly a consequence of the formation of DNA photoproducts such as cyclobutane-pyrimidine dimers [76].

Exposure to UVA and UVB causes degeneration of the extracellular matrix (ECM), consisting of collagen and elastin, loss of cell viability, membrane damage, and elastosis (elastin/fibrillin deposits) [77,78,79]. ECM degradation is associated with increased expression and/or activity of matrix metalloproteinases (MMPs), primarily MMP-1 cleaving interstitial collagen and MMP-2 degrading the basement membrane and damaged interstitial collagen. MMP-1 and MMP-2 inhibitors include tissue metalloproteinase inhibitors (TIMPs), e.g., TIMP-1 and TIMP2 [80,81,82].

Photoaging and the development of skin diseases, including cancer, are inextricably linked to the remodeling of the ECM as a result of increased expression of MMPs or decreased expression of TIMPs under the influence of oxidative stress. There is no doubt that high-energy, short-wave radiation (UVB and UVA) causes oxidative stress [83]. Almost half of free radicals are formed as a consequence of exposure to sunlight at ~360 nm [84]. Therefore, the preventive factor for the above dysfunctions is therapy with compounds with antioxidant properties.

Zastrow et al. [85] using quantitative ESR x-band spectroscopy determined the free radical threshold value (FRTV) to be approximately 3.5 × 10^12^ radicals/mg of tissue, which represents the ratio of reactive oxygen species (ROS)/secondary lipid oxygen species (LOS) in skin. Under stress conditions using ex vivo human skin irradiated with ultraviolet + visible light (UV + VIS), UVB + UVA, and VIS, the favorable ratio of ROS > LOS changes to LOS > ROS. Reversal of the physiological ROS/LOS relationship in the skin is a sign of imbalance in the redox system and the possibility of harmful effects (distress).

Also, Lohan et al. [86] confirmed the LOS formation as a consequence of UVA-LED (365 ± 5 nm) in situ-irradiation in ex vivo porcine skin applying x-band electron paramagnetic resonance (EPR) spectroscopy with quantification with the spin probe 3-(carboxy)-2,2,5,5-tetramethylpyrrolidin-1-oxyl (PCA) and the spin trap 5,5-Dimethyl-1-Pyrroline-N-Oxide (DMPO). Moreover, the authors attest to the skin integrity and viability of the skin cells by using an MTT [3-(4,5-dimethylthiazol-2-yl)-2,5-diphenyltetrazolium bromide] assay during the experiments. The key point was within 0.5 MED, where LOS increases as ROS decreases. Dose determination was possible thanks to in-situ irradiation using an LED diode. In previous studies using cut models of animal skin or reconstructed models of human skin, the power of solar simulators, i.e., fiber-coupled solar simulators, was too low [87].

Albrecht et al. [88] claim that it is more advantageous to use only one spin marker for the quantitative determination of radicals because PCA and PBN give comparable quantitative results for the detection of ROS and LOS radicals. Furthermore, the use of a single spin marker in the assays prevents inter-individual variability and interference due to artifacts that occur over time.

### 3.2. Infrared Radiation (IR)

IR radiation is perceived primarily as heat, causing tissue heating, vasodilation, erythema, thermal pain, and circulatory collapse [89].

To assess the effect of IR radiation on the skin, a unit called the minimum heating dose is used, defined as the radiation dose needed to constantly increase the temperature of the skin exposed to IR radiation with an intensity of 2.02 W cm^−2^ with maximum emission at 1100–1200 nm [90]. The authors of this study reached a temperature plateau after 652 ± 22 s. They also noticed that the value of the minimum heating dose is directly proportional to the radiation intensity.

Research over the last decade has shown that IR radiation is associated with photoaging of the skin [6,91,92,93,94,95,96,97,98,99]. IRA-induced photoaging involves the induction of MMP-1 without the induction of its inhibitor, TIMP-1, resulting in collagen degradation [91,92]. Increased expression of matrix metalloproteinases and decreased activity of antioxidant enzymes cause wrinkles [100]. Disturbances of electron flow in mitochondria caused by IR have also been detected, resulting in impaired energy production in fibroblasts and modulation of mitochondrial signaling pathways [91]. IR has also been shown to stimulate angiogenesis and increase the number of mast cells, which is associated with skin photoaging [91].

In turn, studies on the effect of IR on oxidative stress are inconsistent, with both no effect and a decrease in the content of antioxidants or free radicals in human skin [6,99] after exposure to IRA [92,98]. Some in vitro studies performed using human skin fibroblasts indicate the protective effect of IR radiation against cytotoxic substances and DNA damage induced by UVA and UVB [95,96,97]. Recent in vitro studies on human fibroblasts have linked the thermal effect with the generation of free radicals. It turned out that in this case, below 37 °C, IR does not generate free radicals; at higher temperatures, free radicals are induced [101].

### 3.3. Visible Light (VL)

Visible light in high doses (VL) (400–760 nm) can also cause skin erythema [102]. In the case of darker skin types IV–VI (Fitzpatrick classification of skin types), greater heat production occurs under the influence of IR, which results in vasodilation and the appearance of erythema, which increases with increasing VL doses. In the case of type II skin, erythema is possible when the light source also emits UVA, while VL itself does not cause erythema or significant pigmentation [103]. VL at doses greater than 720 J cm^−2^ can induce skin pigmentation in the absence of significant UVR radiation [100]. Other studies show that even at a much weaker exposure of 45 J cm^−2^, immediate pigment darkening (IPD) was observed in the wavelength range from 380 to 500 nm [104]. The inconsistency of results between individual studies results from the use of different light sources and different doses applied to the skin of the subjects [102,105].

The use of sunlight with filters (<400 nm) causes more intense so-called permanent pigment darkening (PPD) and IPD than those induced by VL (>420 nm) [105]. Studies using a solar simulator (385–690 nm) in people with skin types II–IV showed that the threshold dose for IPD is 40–80 J cm^−2^, and for PPD it is greater than 80 J cm^−2^ [103]. Type IV–VI skin pigmentation induced by VL was darker and more persistent in darker skin types even compared to UVA-induced pigmentation [102]. The authors proved that VL contributes to the transfer of melanin from the basal layer to the upper layers of the epidermis, which was demonstrated using confocal microscopy and diffuse reflectance spectroscopy methods. The ability of VL to induce pigmentation explains the involvement of VL in the pathogenesis of pigmentation disorders often occurring in darker skin types, e.g., melasma or post-inflammatory hyperpigmentation [106].

## 4. Application of Carotenoids for Skin Photoprotection

Carotenoids belong to a large group of natural pigments commonly found in nature. They occur not only in plants, but also in photosynthetic bacteria, some species of archaea, fungi, algae, and animals. About 1200 different carotenoids have been described so far, each of which can form several cis-trans isomers [107]. The list of discovered carotenoids is constantly growing and is available on the website (http://carotenoiddb.jp, accessed on 15 June 2015), which is constantly updated. The last update performed on 1 November 2020 identified 1204 natural carotenoids in 722 source organisms.

**Figure 6:**
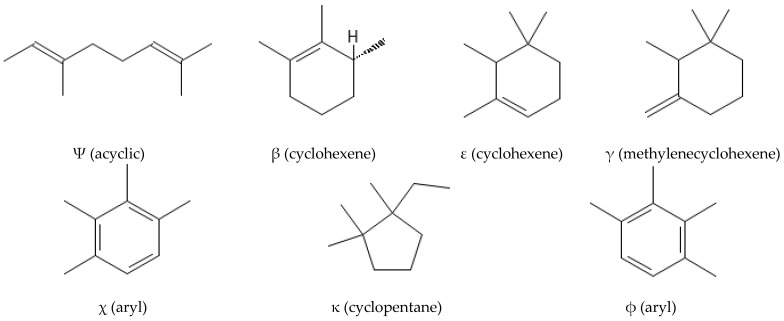
Terminal rings of carotenoid molecules.

From a chemical point of view, carotenoids are tetraterpene compounds mostly composed of several isoprene units. There are usually nine conjugated double bonds in the end-group terminated carbon skeleton (Figure 6) [108], C-40, C-45, or C-50 in the case of higher carotenoids, or C < 40 in the case of apocarotenoids. Carotenoids are basically divided into two groups, i.e., carotenes, which are hydrocarbons, and xanthophylls containing oxygen atoms, which can come from various functional groups, i.e., –OH (hydroxyl), =CO (carbonyl), –CHO (aldehyde), –COOH (carboxyl), epoxy, and furanoxide [109].

The color of carotenoids is the result of light absorption, which is possible due to the presence of an extensive system of conjugated double bonds [109]. The first studies confirming the photoprotective effect of carotenoids on humans were carried out in the 1970s [110,111,112]. Many studies were performed on mice in the 1980s, which confirmed that a diet rich in carotenoids reduces the incidence of skin cancer [113,114,115]. So far, only 25 human clinical studies have examined the photoprotective effects of carotenoids on the skin [116]. In the Pubmed database for the years 1982–2023, there were 24 clinical trials, meta-analyses, and randomized clinical trials, of which only 14 met the inclusion criteria (Table 3). The skin’s susceptibility to sunburn was most frequently assessed. erythema caused by UVB radiation. In recent years, more attention has been paid to the photoprotective role of carotenoids against UVA radiation in human skin. These include their effects on oxidative stress markers such as intercellular adhesion molecule 1, heme oxygenase-1, interleukins, and matrix metalloproteinases [117,118,119,120]. These carotenoids have been shown to have a protective effect against oxidative damage by inhibiting the decline in antioxidant enzyme levels in UVA-exposed cells, reducing the levels of UVB-induced oxidative stress metabolites (i.e., malondialdehyde and 4-hydroxyalkenals).

The bioavailability of carotenoids depends on the type of food matrix and connections with other biomolecules, e.g., proteins [134]. Increased temperature used in food preparation has been shown to increase the bioavailability of carotenoids by destroying cell walls [135,136]. In turn, poor health, especially abnormalities in fat absorption, reduces the absorption of carotenoids, as do interactions with some drugs such as sulfonamides and aspirin [137]. Carotenoids are distributed in the blood in the form of very low-density lipoproteins (VLDL) [138,139,140,141,142,143,144,145,146]. Nearly 20 carotenoids have been found in human blood, including β-carotene, α-carotene, lycopene, lutein, zeaxanthin, β-cryptoxanthin, α-cryptoxanthin, γ-carotene, neurosporene, ζ-carotene, phytofluene, and phytoene [140,143]. UVR, through the formation of ROS, reduces the level of carotenoids in the plasma [144] and in the skin in humans by 13.5% and 21.2% (*p* < 0.05) after exposure to blue and violet light (50 J/cm^2^ and 100 J/cm^2^) [145]. A reduction in carotenoid concentration was observed in basal cell carcinomas and actinic keratosis [146].

**Figure 7:**
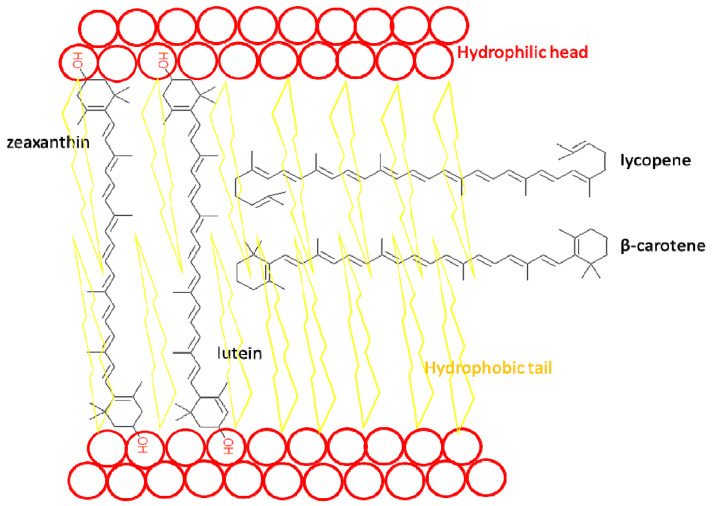
Localization of carotenoids within the phospholipid bilayer.

Carotenoids accumulate in the epidermis and dermis, so they play an important role in the skin’s antioxidant defense. Due to their significant lipophilicity, they localize within cell membranes. Inside the lipid bilayer, carotenoids assume different spatial orientations (Figure 7). While β-carotene and lycopene are arranged along the membrane, more polar molecules containing oxygen atoms, such as lutein and zeaxanthin, are perpendicular to the membrane surface [147].

The highest concentration of carotenoids occurs in the subcutaneous tissue, where they accumulate in adipocytes [148,149] and in lipid plaques in the SC [150]. Carotenoids are not evenly distributed in the SC. The highest concentration is on the surface and near the bottom, where they accumulate through keratinization and blood circulation [9,10,151,152,153]. The review by Darvin et al. was devoted to the study of carotenoid kinetics in human SC in vivo using non-invasive optical and spectroscopic methods, which include resonance Raman spectroscopy (RRS), confocal Raman micro-spectroscopy (CRM), skin color measurements, and diffuse reflectance spectroscopy [154]. Pigment deposition is more visible on the palms and soles of the feet due to the thicker SC layer in these areas [155]. The discolorations characteristic of carotenemia and lycopenia occur as a result of the conversion of accumulated carotenoids to vitamin A [156,157,158,159]. For example, a case was described of a 68-year-old Caucasian woman who had red-orange discoloration of the skin of the hands and soles. It turned out that her diet included 1 kg of kaki fruit per day [157]. Another case of carotenemia caused by excessive β-carotene supplementation was described in a 66-year-old woman [159].

In vitro studies using cultured human skin fibroblasts demonstrated the antioxidant properties of carotenoids, i.e., lycopene, β-carotene, and lutein, which effectively remove peroxide radicals and free oxygen radicals (ROS) and inhibit the peroxidation of lipids produced by exposure to UVR radiation [160,161,162,163,164,165,166]. A 2002 study noted that although all carotenoids provide a protective effect, the amounts needed for this purpose varied between substances. The most effective carotenoid turned out to be lycopene, the amount of which was the smallest to ensure effective photoprotection against UVB and amounted to 0.05 nmol/mg of protein. The remaining carotenoids tested, β-carotene and lutein, were less effective; hence, the amount needed for photoprotection was many times higher and amounted to 0.40 and 0.30 nmol/mg of protein, respectively. It should be noted that a further increase in the level of carotenoids led to a pro-oxidant effect [167]. The effectiveness of carotenoids in preventing lipid peroxidation and inducing DNA damage caused by oxidative stress is also confirmed by other studies [168,169]. Most research on the photoprotective effect of carotenoids concerns β-carotene. Many of them have been performed in vivo with humans [111,123,170,171,172,173,174,175]. Due to the fact that doubts have arisen regarding the safety of the use of beta-carotene, especially the incidence of lung cancer [176], other carotenoids are increasingly used for supplementation, e.g., lycopene [177,178] or mixtures containing carotenoids, e.g., 8 mg of β-carotene, lycopene, and lutein balances 24 mg of β-carotene regarding the level of photoprotection [172]. The advantage is that some carotenoids, e.g., lutein and zeaxanthin, are more resistant to degradation than, e.g., β-carotene and lycopene. Combinations of carotenoids with other antioxidants may act synergistically, such as combinations of carotenoids with vitamin E [174], polyphenols [179], lycopene, β-carotene, and vitamins C and E [180,181,182]. It should be noted that, as shown, supplementation with natural preparations, e.g., tomatoes rich in lycopene, provided better photoprotection compared to synthetic lycopene [127]. The photoprotective effect of colorless tomato carotenoids, phytoene and phytofluene, containing fewer than colored carotenoids (11 conjugated double bonds), and 3 and 5 double bonds, respectively, was also reported in in vitro studies [183].

Carotenoids act as filters for blue and near-ultraviolet light. The antioxidant properties of carotenoids are related to their ability to quench singlet molecular oxygen (^1^O_2_) and other reactive oxygen species (lipid peroxides, superoxide anions, hydroxyl radicals, or hydrogen peroxide) [184,185,186]. Thanks to this, carotenoids protect against reactions including DNA damage and lipid peroxidation. Carotenoids can quench singlet oxygen, which finally leads to energy dissipation as heat, thanks to low-lying triplet states. The triplet state of carotenoids (^3^CAR) is produced electronically by energy transfer, which was first demonstrated by Foote and Denny [187].

^1^O_2_ + ^3^CAR → ^3^O_2_ + CAR + heat (1)

Reactions of carotenoid with free radicals involve adduct formation (2), electron transfer (3), and hydrogen atom transfer (4).

(2) $$R^{\text{●}}+\mathrm{CAR}\;\to {\mathrm{R-CAR}}^{\text{●}}\;\overset{R^{•}}{\to }\mathrm{R-CAR-R}$$

R^●^ + CAR → R^−^ + CAR^●+^ (R^+^ + CAR^●−^) (3)

R^●^ + CAR → RH + CAR^●^ (4)

The products of these reactions may be potentially pro-oxidant factors, e.g., carotenoid radical cations (CAR^•+^) [188,189], which in vivo have the ability to oxidize amino acids, changing the functionality of proteins [190]. Using the example of astaxanthin, it was shown that the oxidative potential of carotenoids may change in the presence of salt [191]. Furthermore, carbon-centered radicals react with oxygen to form peroxy radicals (5). Thus, the antioxidant properties of carotenoids depend on the oxygen concentration [192].

(5) $${\mathrm{CAR}}^{\text{●}}+O_{2}\;\to {{\mathrm{RO}}_{2}}^{\text{●}}$$

The ability to quench singlet oxygen depends on the number of conjugated double bonds. Carotenoids with the number of conjugated double bonds n < 5 showed no quenching with the quenching rate constant *k*_q_ < 0.01 × 10^9^ M^−1^ s^−1^, whereas polyenes with n = 9 were the most efficient quenchers with *k*_q_ < 16 × 10^9^ M^−1^ s^−1^ [193]. The two carotenoids accumulating in the eye, zeaxanthm and lutein, possess unespecified and varied *k*_q_ values. Zeaxanthin with n = 11 (*k*_q_/10^9^ Ms = 12.6 in benzene) is twice as effective as lutein with n = 10 (*k*_q_/10^9^ Ms = 6.64 in benzene) [184]. It may indicate different actions of these carotenoids in the protection of the eye.

The importance of carotenoids in preventing sunburn has been known for many years [111,169,170,173,174,194]. However, the photoprotective effect of carotenoids in the development of non-melanoma skin cancer initiated by UVR is not elucidated. Some studies question the role of carotenoids in this process [195,196,197]. While others describe the relationship between basal cell carcinoma (BCC) and antioxidant nutrients, specifically the carotenoids vitamin E and selenium, in case-control studies, an example is a study involving 180 participants, which undoubtedly confirmed the existence of a relationship between the risk of BCC and lutein intake [198]. Similar controversies exist in the assessment of the importance of carotenoids in skin photoaging, which manifests itself in the form of skin dryness, lack of elasticity, wrinkle formation, skin tone, additional pigmentation, and telangiectasia. Some studies confirm this relationship [130], while other studies question the existence of a relationship between dietary carotenoid intake and the severity of skin photoaging [154,192,199].

### 4.1. Carotenes

#### 4.1.1. Lycopene

**Figure 8:**
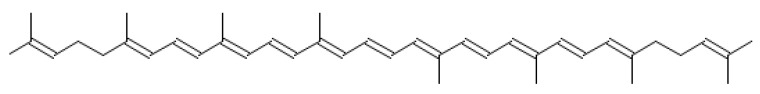
Chemical structure of all-*trans*-lycopene.

Tomato seeds are a rich source of lycopene (Figure 8) [200]. Therefore, following a tomato-based diet is useful for protection against UVR [201]. It should be noted that *trans*-lycopenes are more effective in quenching ROS, e.g., ROO^•^, than *cis* isomers [202].

Protection against burns is provided by lycopene supplementation, which has been repeatedly demonstrated in in vivo human studies [122,203,204]. Randomized control studies conducted in vivo in humans confirmed the effectiveness of lycopene administered as a dietary component in the form of tomato puree (40 g/16 mg of lycopene per day with 10 g of olive oil for a period of 10 weeks) in alleviating the formation of erythema on the back after exposure to UVR [177]. After 10 weeks of treatment, erythema on the back of the treated group (n = 9) was 40% less compared to the control group (n = 10) (*p* = 0.02; Wilcoxon–Mann–Whitney test). In another study, in addition to examining the size of the erythema, immunohistochemical analysis of skin biopsies was performed for procollagen (pC) I, fibrillin-1, matrix metalloproteinase (MMP-1), and a quantitative polymerase chain reaction mtDNA deletion of 3895 bp. This study found that supplementation reduced mtDNA deletion (*p* = 0.01) and reduced the increase in MMP-1 (*p* = 0.04) induced by UVR [178]. The combination of lycopene with other carotenoids and Lactobacillus johnsonii also protected humans against UVA-induced polymorphic light eruptions [121].

In a placebo-controlled, double-blind, randomized crossover study, two groups receiving the lycopene-rich tomato nutrient complex (TNC) or lutein diet were assessed for their ability to reduce the expression of genes that are indicators of oxidative stress, photodermatoses, and photoaging (HO1, ICAM-1, and MMP-1 in polymerase chain reaction with reverse transcriptase) induced by UVA/B and UVA1 radiation [205]. This study found that TNC completely inhibited UVA and UVA/B-induced upregulation of heme-oxygenase 1, intercellular adhesion molecule 1, and matrix metallopeptidase 1 mRNA, regardless of sequence (Anova, *p* < 0.05). However, lutein showed much less effect in the second sequence compared to TNC.

Research confirms that lycopene has the potential to prevent skin cancer. Ascenso et al. [202] studied skin cells irradiated with UVB at five different doses of ~75, 150, 200, 225, and 325 mJ/cm^2^. Previously, cells were exposed to lycopene complexed by DM-β-CD (10 μM) for 24 h.

Exposure to lycopene resulted in overexpression of the BAX gene in irradiated cells and a reduction in the number of cells in the G0/G1 phase. The authors of this study described the role of lycopene as “corrective”, depending on the level of photodamage. A study by Cooperstone et al. from 2017 showed that treating Skh-1 mice for 34 weeks with a tomato-rich diet significantly reduced tumor induction by UVB irradiation compared to animals receiving regular food [202].

#### 4.1.2. β-Carotene

**Figure 9:**
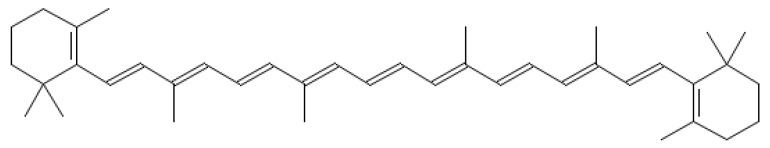
Chemical structure of β-carotene.

In 2008, Köpcke et al. [175] conducted a meta-analysis and showed that β-carotene (Figure 9) supplementation provides protection against sunburn.

Protection required a minimum of 10 weeks of supplementation. Each additional month of supplementation increased this effect by 0.5 standard deviations [175]. Despite the fact that dietary intake of β-carotene reduces UV-induced erythema, this effect is dependent on the dose and duration of supplementation [111,169,171,172,173]. Obtaining a protective effect against UV radiation in humans requires the use of relatively high doses (~≥12 mg/day) for a period of approximately 10 weeks [111,173]. Simultaneously, it has been proven that β-carotene is harmful to people at high risk of lung cancer, e.g., smokers and workers exposed to asbestos. In these cases, the use of high doses of β-carotene significantly increases the risk of lung cancer [175].

Already in the 1980s, in connection with the publication by Burton and Ingold, it was claimed that “β-carotene is not a conventional antioxidant” and maybe a pro-oxidant in an environment of high oxygen partial pressures [206]. Although further research by Jorgensen and Skibsted did not confirm this opinion [207], it was observed that the antioxidant activity of β-carotene increased almost fourfold in a state of hypoxia at 0.01 atm. oxygen. The pro-oxidant properties of β-carotene were observed, in turn, in an air-saturated acetone environment [208]. The pro-oxidant activity of high doses of β-carotene was reported in in vitro studies on a keratinocyte cell line (HaCaT). The authors of this study claimed that the antioxidant activity of β-carotene has been proven to be effective in protecting against UV radiation only at low doses [209].

High doses of β-carotene, 30 to 90 mg/day in children and 60–180 mg/day in adults, are an effective drug for the treatment of photosensitivity in patients with erythropoietic protoporphyria, as confirmed by controlled clinical trials [210]. Recently, *N. Engl. J. Med.* published a report on another effective therapy for this condition involving treatment with afamelanotide, an analogue of the α-melanocyte-stimulating hormone that darkens the skin [211].

The anti-inflammatory activity of β-carotene is also known [212,213]. Oral administration of β-carotene reduced skin inflammation and ECM (collagen, elastin, and hyaluronic acid) degradation, which was confirmed in a study on hairless mice with oxazolone-induced inflammation/oedema. The authors of this study observed an improvement in the barrier functions of the skin after treatment with β-carotene, which reduced inflammatory factors (cytokines: IL-1β, IL-6, IL-4, Par-2, TNF-α, and chemokine: monocyte chemoattractant protein-1 (MCP-1)), promoted the expression of filaggrin (a structural protein in the stratum corneum of the epidermis), and decreased the activity of matrix metalloproteinases (MMPs) (reduced the activity of proMMP-9, but not proMMP-2).

### 4.2. Xanthophylls

About 30% of carotenoids are xanthophylls, the most common of which are lutein, astaxanthin, and zeaxanthin. Studies have confirmed the importance of these compounds in preventing skin photodamage caused by sunlight [130].

#### 4.2.1. Astaxanthin

**Figure 10:**
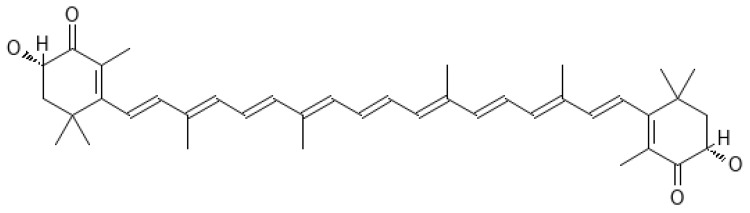
Chemical structure of all-*trans*-astaxanthin.

Astaxanthin (AST 3,3′-dihydroxy-β,β-carotene-4,4′-dione) (Figure 10) is an oxygen derivative of carotenes, a fat-soluble pigment that contains 13 conjugated double bonds.

Naturally, ASTs occur in the form of stereoisomers (3S, 3′S) and (3R, 3′R), geometric isomers, and in free and esterified forms [214]. The ability to biosynthesize AST is possessed by some species of algae (*Haematoccocus pluralis*, *Chlorella zofingensis*), yeast (*Xanthophyllomyces dendrorhous*), bacteria (*Corynebacterium glutamicum*), *Cyanobacteria (Synechococcus* sp., *Agrobacterium aurantiacum, Paracoccus carotinifaciens*, *Escherichia coli*), and Lichene (*Clodia aggregata, Concamerella fistulata, Usnea amaliae, Usnea densirostra*) [215]. AST was detected mainly in fish and crustaceans (lobster, crab, shrimp, salmon, and pink trout) [216,217].

In the human body, AST occurs in combination with the lipoproteins VLDL, LDL, and HDL [218,219]. AST enters the blood and tissues, crossing the blood-brain barrier [220,221]. In vivo studies in rats showed no accumulation of AST in tissues [222,223], and the mean half-life is up to 52 h [219].

The presence of polar functional groups, i.e., ketone and hydroxyl, is responsible for the polarity of this compound and its greater photostability and resistance to light and temperature [220,224,225].

AST has a strong antioxidant effect, scavenging reactive oxygen species (ROS) and reactive nitrogen species (RNS), with a greater antioxidant potential compared to other xanthophylls without pro-oxidant effects. AST has the ability to quench singlet oxygen, which is several times greater than that of β-carotene, and its antioxidant effect compared to vitamin E is up to 100 times stronger in the lipid peroxidation test [226]. AST is 6000 times more potent than vitamin C, 770 times more active than coenzyme Q10 (CoQ 10), 100 times more potent than vitamin E, and 5 times more potent than β-carotene in retaining energy from singlet oxygen [227,228,229].

The effect of the carotenoids β-carotene and AST on liposome peroxidation induced by ADP and Fe^2+^ was investigated. Both compounds inhibited the production of lipid peroxides, with AST being approximately two times more effective than β-carotene. The difference in activity between β-carotene and AST suggests that the effective antioxidant activity of AST is due to the unique structure of the terminal ring moiety [230]. The conjugated polyene moiety and the terminal ASX ring moieties were involved in radical scavenging in the membrane and on the membrane surface, respectively, while in the case of β-carotene, only the conjugated polyene chain was responsible for radical scavenging.

Many human studies have been conducted to investigate the toxicity of AST. Daily administration of 6 mg [231], 4 mg [232], and even 100 mg [218] did not cause side effects. Even doses of 12 g/kg body weight did not produce negative effects in animal studies [233].

The health-promoting properties of AST concern primarily its antioxidant effect and its ability to remove reactive oxygen and nitrogen species [226] and guazine radicals [234]. AST supplementation also increased the activity of antioxidant enzymes (catalase, superoxide dismutase, peroxidase, and TBARS) [235,236].

AST also has anti-inflammatory activity [237]. In an in vitro study using human neutrophils, Rita C. Macedo et al. found the beneficial effect of AST supplementation on the phagocytic and bactericidal abilities in the Candida albicans test, the release of cytokines (IL-6 and TNF-α), the production of reactive oxygen species (superoxide anion, hydrogen peroxide), and nitric oxide (NO^•^), the activity of antioxidant enzymes (Mn-SOD, CAT, GPx, and GR), and oxidative damage (TBARS test and carbonyl groups). The ability of AST to inhibit tumor growth has also been confirmed [238,239].

AST in human skin fibroblasts prevented UVA-induced changes in the activity of antioxidant enzymes, i.e., superoxide dismutase (SOD) and the antioxidant glutathione (GSH) [240].

Studies by Yoshihisa [241] suggest that AST protects the skin against inflammation induced by UVB and UVC radiation. AST caused a reduction in inducible nitric oxide (iNOS) and cyclooxygenase (COX)-2 and decreased the release of prostaglandin E2 from HaCaT keratinocytes after irradiation. AST caused significant inhibition of UV-induced apoptosis, as evidenced by a DNA fragmentation assay. Moreover, the authors of this study point out that AST treatment resulted in a reduction in UVB- or UVC-induced protein and mRNA expression of macrophage migration inhibitory factor (MIF), IL-1β, and tumor necrosis factor α TNF-α in HaCaT keratinocytes. The above observations are also confirmed by other authors [216]. Suganuma et al. [242] observed that AST supplementation before and after UVB and UVA irradiation reduced MMP-1 expression. AST also inhibited UVB-induced AP-1 activator protein expression and reduced UVB-induced phosphorylation of several MAPK family members by transactivating AP-1 in human fibroblasts.

A recent study by Komatsu et al. [216] demonstrated a beneficial effect of oral AST on the prevention of skin photoaging in vivo. In a mouse model, AST inhibited the UVA-induced decrease in the levels of pyroglutamic acid (PCA) and urocanic acid (UCA), which are the main natural moisturizing factors in the epidermis. In this mouse model, AST also inhibited UVA-induced matrix metalloprotease 13 (MMP-13) expression, which may highlight its photoprotective effects against skin photodamage [47].

Chung et al. reported a beneficial effect of AST on human skin aging [243]. The same group of researchers is conducting a clinical trial to determine the effect of AST or isoflavone supplementation on skin elasticity, epidermal hydration, and changes in skin barrier integrity. However, the results of this study are not yet available.

Many preclinical and clinical studies confirm that AST has a positive effect on skin health [244], i.e., preventing UV-induced photoaging. This was confirmed in a study on mice in which a reduction in transepidermal water loss associated with exposure to ultraviolet radiation, a reduction in the expression of aquaporin 3 and other proteins were observed [216], and an increase in the number of collagen fibers were observed [245]. Human studies supplemented with a dose of 6 or 12 mg/day observed prevention of age-related skin damage and improvement of skin conditions by preventing the secretion of inflammatory cytokines from keratinocytes and reducing the secretion of matrix metalloproteinase-1 by dermal fibroblasts [246]. Studies on humans supplemented orally with 4 mg/day [247] confirmed the antioxidant effect and facial skin rejuvenation. Reduction of the skin damage caused by exposure to UV rays was provided by similar supplementation with AST 4 mg/day orally in human studies [248].

Many experiments have demonstrated the inhibitory effect of AST on UVR-induced cytotoxicity and epidermal keratinocyte cell death [216,217,245,246,249,250,251,252].

#### 4.2.2. Lutein

**Figure 11:**
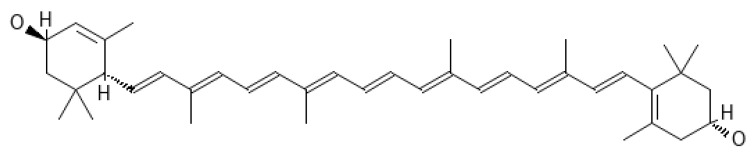
Chemical structure of all-*trans*-lutein.

Lutein (Figure 11) is abundant in dark green leafy vegetables, and next to zeaxanthin, it accumulates in the macula of the human retina and the skin [253].

Lutein, like other xanthophyll carotenoids, has antioxidant and anti-inflammatory properties and prevents photodamage caused by UV radiation. From a chemical point of view, lutein is the dihydroxyl form of α-carotene. The absorption maximum for lutein is 445 nm. Thanks to this, lutein filters radiation in the range of 400–475 nm (blue light), protecting, among others, the retina and skin against oxidative damage [254].

Lee et al. conducted studies on mice, which confirmed that orally supplemented lutein accumulates in mouse skin and inhibits ROS production after exposure to UVR radiation [255]. Moreover, lutein significantly reduced the adverse effects that UVR (280–320 nm, UVB) has on the skin, i.e., tissue edema and contact hypersensitivity (CHS) in the low-dose model. This study authors noted that no effect was observed in the high-dose UV-induced immunosuppression model.

The beneficial effect of orally administered lutein and zeaxanthin on the harmful effects of UVB radiation is also confirmed by another study from 2003 [256]. In a case-control study, it was shown that reducing dietary lutein intake increased the risk of melanoma [257].

Astner et al. extended their studies to chronic photodamage (UVB with a total dose of 16,000 mJ/cm^2^) and photocarcinogenesis (30,200 mJ/cm^2^) and observed reduced UVB-induced inflammatory responses in hairless mice (Skh-1) supplemented with 0.4% lutein and 0.04% zeaxanthin. These photoprotective effects included a reduction in skinfold thickness and the number of infiltrating mast cells. In the case of photocarcinogenesis, increased tumor-free survival, decreased tumor multiplicity and total tumor volume, and lower numbers of bromodeoxyuridine and proliferating cell nuclear antigen (PCNA)-positive cells in the epidermis were observed in lutein/zeaxanthin-treated mice compared to controls [258].

Phillips et al. (2007) analyzed the protective effect of lutein on the ECM. The expression of elastin, MMP-1, MMP-2, TIMP-1, and TIMP-2 was examined in skin fibroblasts and melanoma cells irradiated with UVA or UVB rays. The authors of this study, conducted in vitro on cell cultures of dermal human fibroblasts and melanoma cells, showed that lutein inhibits cell loss, membrane damage, and elastin expression and increases cell viability. This study confirmed that lutein is responsible for the inhibition of MMP-1 and MMP-2 overexpression induced by UV radiation and the stimulation of TIMP-2 [259,260].

In a placebo-controlled, double-blinded, randomized, crossover study, it was shown that orally supplemented lutein reduces the overexpression of genes, i.e., the oxidative stress indicator gene HO1 (heme-oxygenase 1), the ICAM-1 (intercellular adhesion molecule 1) gene, which is increased in expression in damaged skin with polymorphic light eruption (PLE), and MMP-1 (matrix metallopeptidase 1), which is responsible for the breakdown of collagen, induced by UVA and UVB radiation [205]. This study involved 65 healthy volunteers, of whom one therapeutic group received the lycopene-rich tomato nutrient complex (TNC) and the other received lutein. The authors of this study emphasized that while TNC completely inhibited gene expression induced by UVA1 and UVA/B (ANOVA, *p* < 0.05), lutein had a photoprotective effect only when taken at the early stage of the experiment.

#### 4.2.3. Zeaxanthin

**Figure 12:**
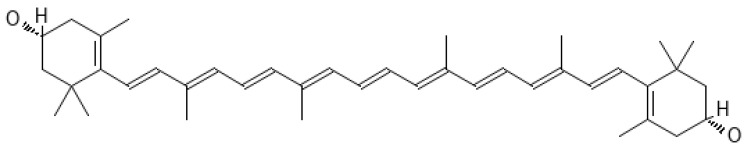
Chemical structure of all-*trans*-zeaxanthin.

Zeaxanthin (Figure 12) is a pigment specific to maize (maize); it gives bright colors to fruits and vegetables; it is found in legumes, seafood, and eggs; but it is also produced by many bacteria [261,262].

Zeaxanthin, together with lutein, are the so-called macular carotenoids present in the macula of the retina. Zeaxanthin, like lutein isomers, filters blue light, can block the formation of melanin pathways, reduce the level of cytokines, and increase the level of antioxidants, protecting against photodamage. Zeoxanthin exists in the form of isomers (3R, 3′R)-zeaxanthin and (3R, 3′S)-meso-zeaxanthin, which differ in the position of a single double bond. On an industrial scale, extracts from marigold flowers (*Tagetes erecta* L.) are used to produce lutein/zeaxanthin supplements.

Isomers of zeaxanthin, similar to lutein, inhibit the peroxidation of membrane lipids and suppress singlet oxygen [263,264]. In vitro studies using human lens cells and rat kidney fibroblasts demonstrated the photoprotective effect of a mixture of lutein and zeaxanthin [258,265,266].

So far, experiments have been carried out on animals, e.g., mice, which have shown that the use of a diet enriched with 0.4% or 0.04% lutein and zeaxanthin reduces skin inflammation caused by UVB, decreases the number of apoptotic cells, and increases cell proliferation [256].

There are several human clinical trials investigating the beneficial effects of lutein and zeaxanthin supplementation on reducing oxidative stress and UVR-induced skin damage [130,267,268,269]. The study by Juturu et al. [270] assessed the photoprotective effects of lutein supplementation (10 mg/d) and zeaxanthin isomers (2 mg/d) administered with the diet in the form of an oil suspension (Lutemax^®^ 2020 soft gelatin capsules). This study was a randomized, double-blind, placebo-controlled study conducted on a population of 50 adult men and women. As a result of the therapy, an improvement in the condition of the skin was achieved (skin brightening, elasticity, and greater firmness), and a phoroprotective effect was visible in the form of a reduced intensity of erythema caused by exposure to UV light. The skin-brightening effect is the result of limiting the formation of two types of melanin (pheomelanin and eumelanin).

#### 4.2.4. Bacterioruberin

**Figure 13:**
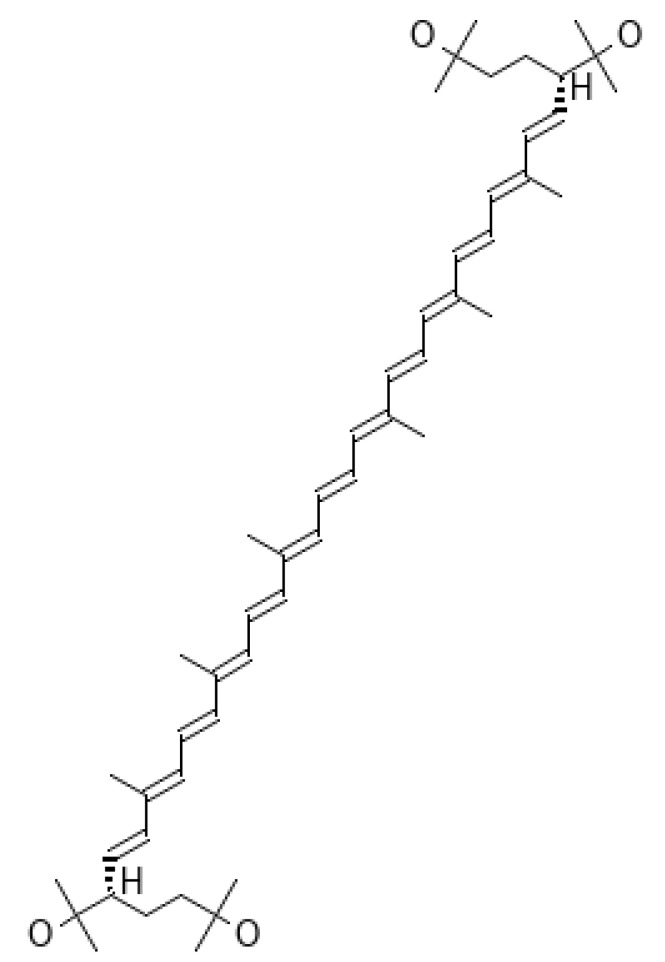
Chemical structure of bacterioruberin.

Bacterioruberin (Figure 13) is a red xanthophyll pigment produced by the microorganisms halophilic archaea, halophilic Haloarchaea such as *Halobacterium salinarium*, *H. mediterranei*, *Haloferax volcanii*, *H. cutirubrum*, and *Halorubrum tebenquichense*, bacteria such as *Rubrobacter radiotolerans*, and psychrophiles such as *Arthrobacter agilis* and *A. bussei*, which occur in saline waters and desert areas with 2.5–5.2 M NaCl [271,272,273,274,275,276,277].

These species are very radioresistant, with cell resistance to freeze-thaw stress [278,279]. This pigment has many functions, ensuring survival under high osmotic and oxidative stress [280], including protection against UV light [281,282], increasing the hydrophobicity of the cell membrane, minimizing intracellular water loss, and allowing oxygen molecules to pass through the cell membrane.

In 2023, Noby et al. [283] described the economic production of carotenoid bacterioruberin C50 and its derivatives from psychotrophic bacteria of the *Arthrobacter agilis* NP20 strain.

The use of bacterioruberin as a UV filter is difficult due to its high molecular weight (500 Da), which impairs skin penetration in vivo [284,285]. However, there are attempts to use haloarchaeal extracts as protective agents against radiation-induced skin damage. In 2014, an American patent was filed for a topical Halobacteria Extract Composition intended for the regeneration of skin tissue [286].

Recent studies conducted in vitro on cell lines have shown that haloarchaeal carotenoids exert anticancer effects in colorectal, breast, liver, and cervical cancer [287]. Giani et al. (2023) proved that Haloferax mediterranei bacterioruberin-rich carotenoid extracts exert selective antiproliferative and cytotoxic effects on human breast cancer cell lines in comparison to a healthy mammary epithelium cell line [288]. The results appear to be very promising considering that the triple-negative BC subtypes, namely MDA-MB-231 and MDA-MB-468, exhibit very aggressive behavior.

An extensive review by Morilla et al. from 2023 [217] collected current preclinical research on the treatment of inflammatory diseases using nanomedicines containing AST and bacterioruberin.

## 5. Nanotechnology-Based Carotenoid Delivery Systems

Compared to xanthophylls, carotenoids have a very labile structure that is susceptible to degradation under the influence of heat, light, oxygen, and catalysts [289,290]. The presence of unsaturated bonds in the hydrocarbon chain causes these compounds to easily undergo oxidation, hydrolysis, and isomerization reactions during extraction and storage [289]. The bioavailability of carotenoids from natural sources is also very low and ranges from 5 to 30% [291,292,293]. Such a low degree of bioavailability results from the fact that in plants, carotenoids are located together with chlorophylls in chloroplasts and chromoplasts in the form of pigment-protein complexes [294]. Another limitation regarding carotenoids is, in addition to chemical instability, low bioavailability, low solubility, and tissue permeability. For example, lycopene is highly lipophilic (log*P* ~ 15). This feature causes lycopene to remain in the stratum corneum and not penetrate the deeper layers of the skin [295]. Poor solubility and permeability through the stratum corneum of phytochemicals result in their poorer bioavailability. 

To facilitate bioavailability and protect the structure against enzymatic decomposition and physicochemical degradation, carotenoids are transformed into so-called nanotechnology products, or “controlled release systems”, i.e., liposomes, micelles, lipid nanoparticles, polymer micro-, and nano-particles or nanocapsules [296,297,298,299,300,301]. Thanks to appropriate nanoencapsulation, nanosystems are protected against degradation and ensure active substance release control [202,295,302,303,304,305]. Despite these undeniable advantages, some reservations are raised by the tendency of nanoparticles to agglomerate, potential toxicity, possibility of bioaccumulation, and production costs [306]. To evaluate topical nanotechnology preparations, appropriate in vitro safety tests are used in the form of 3D EpiSkinTM^®^ and EpiDermTM^®^ skin models and 2D in vitro models, e.g., the human keratinocyte cell line HaCaT and the BALB/c 3T3 mouse embryonic fibroblast cell line, along with the MTT test [307].

Nanoformulation systems can be constructed based on inorganic materials (metallic nanoparticles) and organic materials (lipids and polymer nanoparticles) [308]. Nanoparticle carriers should be biocompatible and biodegradable [309,310]. It has been shown that carbon nanoparticles, metallic nanoparticles, and even solid lipid nanoparticles (SLN) and nanostructured lipid carriers (NLC) may be potentially toxic [307] due to the small size or composition of the nanocarriers. A method to avoid skin irritation and other adverse effects on the immune system is the use of biocompatible excipients and an appropriate encapsulation strategy. Nanoencapsulation of bioactive compounds and drugs using lipid-based systems is popular in the pharmaceutical and food industries.

In 2013, Ascenso et al. [311] created vesicular nanocarrier formulations for dermal delivery of lycopene. The improvement of lycopene bioavailability through the skin was studied in vitro using a human keratinocyte cell line and in vivo with mice. The advantage of nanoformulations over conventional products in repairing UVR-damaged keratinocytes and pre-cancerous conditions was confirmed. Various formulations have been developed to increase the stability and bioavailability of β-carotene and astaxanthin in the form of nanoemulsions (NE), hydrogels/lipogels, liposomes, and NLCs.

A few reviews have been published on the progress of new nanoformulations containing plant extracts for topical application to the skin [312,313]. Nanoencapsulation systems containing natural bioactive compounds were collected, among others, by Taouzinet et al. [314]. Table 4 summarizes examples of various nanoformulations containing carotenoids to increase stability and bioavailability, either for topical or oral delivery.

Liposomal preparations containing phospholipids, which improve the solubility of carotenoids in water, deserve attention. The use of a high-pressure homogenizer (MicrofluidizerTM) made it possible to obtain nanosystems with a diameter of less than 100 nm [319], which structurally resemble the cell membrane and can connect to the stratum corneum [351]. Zhao et al. [352] developed nano-liposomes of lycopene with increased stability, which was confirmed in in vivo tests on an animal model. During in vivo studies, it turned out that lycopene encapsulated in nanoliposomes improves renal dysfunction [353]. The combination of doxorubicin and lycopene-loaded liposomes demonstrated enhanced antitumor efficacy in in vivo studies in B16 melanoma mice [354].

Another nanomaterial is polymer nanofibers, mainly polycaprolactone (PLC), which show good tissue biocompatibility and gradual drug release [355]. High production rates of nanofibers are achieved using electrospinning [356]. This method was used to produce β-carotene-NF with a diameter of 400–800 nm [316].

Solid lipid nanoparticles (SLN) have been investigated for their suitability for carotenoid processing [330]. SLN is most often obtained using the high-pressure homogenization method. In addition to the lipid with the active substance, the creation of a stable dispersion in the aqueous phase requires the presence of a surfactant and a co-surfactant [357]. It has been noticed that high-melting-point (HM)-lecithin is very useful as an antioxidant and stabilizer of the lipid carrier system. Moreover, the stability of colloidal dispersions and the increase in β-carotene uptake in Caco-2 cells are significantly enhanced when a layer of phosphoserine, whey protein isolate, and especially sodium caseinate is formed at the oil-water interface [329].

Oil-in-water nanoemulsions (NE) consist of small lipid droplets between 10 and 200 nm dispersed in an aqueous phase. Fathi et al. [358] described methods for producing NE. The most frequently used methods are high-energy methods (increased pressure, homogenization, microfluidization, and ultrasound). Among the low-energy methods, the author mentions solvent diffusion. Combined methods, such as high-shear mixing, can also be used. NEs ensure small droplet sizes, but the stabilization of an emulsion containing carotenoids requires many additives [328]. The selection of the emulsifiers that reduce interfacial tension, i.e., polyoxyethylene sorbitan esters of fatty acids, amphiphilic proteins, phospholipids, or polysaccharides, appears to be very important. NE loaded with carotenoids extracted from sweet potato peel with an average particle size of 15.7 nm was prepared with the addition of Tween 80, PEG 400, soybean oil, and deionized water [326]. Of the dozen or so carotenoids detected, all-trans-β-carotene was the most abundant. The obtained NE proved effective in inhibiting tumor growth in mice in vivo and in breast cancer cell lines (MCF-7). An antiproliferative effect was observed, reducing the levels of epidermal growth factor (EGF) and vascular endothelial growth factor (VEGF). In another study, stable oil-in-water NE with a droplet size (<150 nm) loaded with carotenoid was obtained using whey protein, gum arabic, and soy lecithin [327]. Preparation of oil in water NE loaded with lycopene requires the use of stabilizers, i.e., medium-chain triglycerides and starch modified with octenyl succinate anhydride (OSA) [323], oil phases, e.g., sesame oil, linseed oil, or walnut oil, and an emulsifying agent, e.g., lactoferrin [324].

Nanostructured lipid carriers (NLC) are suitable for transdermal administration and, unlike solid lipid nanoparticles (SLN), improve drug photostability, improve drug release, increase drug absorption through the skin, and reduce the risk of systemic side effects and irritations [359,360,361]. It is known, for example, that the therapeutic potential of lycopene is limited due to its poor stability and low water solubility [305,362]. Stable lycopene-loaded nanostructured lipid carriers (NLCs) were successfully produced by hot high-pressure homogenization (HPH) as local drug delivery systems [321]. It should be emphasized that the therapeutic properties of NLC depend also on the type of surfactants used, i.e., C-1216 (sucrose laurate), C-1816 (sucrose stearate), C-1616 (sucrose palmitate), C-1815 (sucrose stearate), and Plantacare 1200 (lauryl glucoside), which provide stabilization of nanosystems. The most beneficial were Plantacare 1200 and carriers made of orange wax, lycopene oil, and rosemary oil. It turns out that the addition of cholesterol to NLC adversely affects the stability of lycopene [363] in contrast to rice bran oil [344]. Thanks to NLC, the stability of lycopene, as expressed by its half-life, increased approximately 20 times compared to its stability in solution. Most attention has been paid to the creation of nanopreparations of lipophilic β-carotene, which, due to its poor solubility in water, requires the use of special carriers to improve bioavailability [332]. Tested solutions, including β-carotene in lipid carriers, are not always effective in protecting against degradation. The addition of vitamin E proved to be effective, ensuring the stability and protection of beta-carotene against oxidation for a period of about half a year, even after diluting the dispersion [325].

Polymer nanoparticles were used for nanoencapsulation of carotenoids (Table 3). Polymer nanoparticles (nanocapsules or nanospheres) are vesicular systems or spheres made of a polymer membrane in which an aqueous or oil solution of the active substance is trapped. Polymer membrane made of PLA or its copolymers, poly(lactide-co-glycolide) (PLGA) and poly(ε-caprolactone), is usually performed [364].

## 6. Discussion

The impact of UV, VL, and NIR wavelengths on the condition of the skin depends on the dose determined by a combination of parameters, i.e., wavelength, fluence, and radiation intensity. UVR light makes up 2–3% of the solar spectrum, while VL and NIR make up about 45–50% of the solar radiation spectrum. Of the incident radiation, only a dozen or so percent is absorbed in vivo by the dermis. Much attention has been paid to UVR, which is believed to be responsible for photoaging and skin cancer [365], while research on the impact of NIR has recently appeared [56]. It is currently believed that exposure to VL and NIR is beneficial to the skin and even necessary to protect the skin from UVR, but there is evidence that NIR damages collagen in the skin and increases MMP-1 activity.

The problem in objectively assessing the results of studies of various groups, or in vivo/in vitro correlations, is the type of light source used in this study, which could reproduce solar radiation [365,366,367,368,369]. The authors of this research point out that in in vitro studies, the radiation intensity is unnaturally high and reflects conditions of rather extreme exposure, e.g., in sunscreen tests [280,365]. An example is Hydrosun 500 [370], a pulsed high peak power broadband (IPL) from Cutera [371], or Infrared-300 by Daekyoung [372] using high levels of irradiation. Therefore, there is a contradiction regarding, for example, the effect of NIR, in which in vitro studies induce the expression of MMPs [93], while in vivo studies accelerate wound healing [373]. Currently, spectra reflecting real solar exposure conditions are available on the website of the National Renewable Energy Laboratory [374].

Another important issue from the point of view of research on the activity of carotenoids is the chemical nature of these compounds, which are very unstable and susceptible to degradation under the influence of air and light, as well as their high lipophilicity. For this reason, in vitro studies may be difficult and lead to variable test results. This is the case with lycopene, which does not dissolve in cell culture medium [375,376]. The addition of a solution prepared with an organic solvent, often toxic, such as tetrahydrofuran, to an aqueous solution often causes precipitation [377]. It is possible to use appropriate carriers in cell cultures, such as liposomes, micelles, microemulsions, beadlets, etc., but it should be taken into account that they will affect cellular uptake [201,376]. According to some researchers, the best carriers of carotenoids are niosomes [377]. In turn, Pfitzner et al. [376] demonstrated the usefulness of methyl-β-cyclodextrin (M-β-CD) for the solubilization of carotenoids in in vitro studies. The use of this β-CD derivative ensured stability and repeatability and was not a source of cytotoxicity. Another study from 2016 [201] for this purpose uses another β-CD derivative, dimethyl-β-CD (DM-β-CD), to solubilize lycopene used for sensitization of the nontumorigenic keratinocyte cell line HaCaT subsequently exposed to UVB.

Treatment of skin cancer must include the inhibition and elimination of damage caused by UVR, as well as the formation of cells with cancer potential [378]. Plant-derived products such as carotenoids are studied primarily as photodamage preventers, thanks to their anti-free radical activity, and as repair substances [375,379]. Many in vitro studies use epidermal keratinocyte lines, which are more susceptible to UVB-induced apoptosis than fibroblasts; at the same time, due to their high proliferative capacity and the ability to repair DNA damage caused by UVR, keratinocytes may be more resistant to UV-B radiation [380].

The skin has antioxidant capacity, which makes it photoprotective against moderate exposure to UVR. Phytonutrients are able to increase the innate protective potential of the skin, protecting it against damage. This applies not only to flavonoids and polyphenols but also to carotenoids, which in the plant world provide photoprotection and protection against oxidative stress. Research on the activity of carotenoids has been conducted for over 30 years. Although their photoprotective effect against UVB and UVA and healing potential against skin cancer have been confirmed in human clinical trials and in vitro studies, carotenoids still function as dietary supplements. However, recommended intake doses for these compounds have not yet been established by the agencies regulating nutritional issues in Europe and the USA (the European Food Safety Authority (EFSA) and the US Food and Nutrition Board). The exception is IQQU ADVANCED SUNSCREEN SPF 50, which contains Lycopene Extract in addition to other ingredients, i.e., octyl methoxycinnamate, Simmondsia chinensis (jojoba) seed oil, titanium dioxide, tocopheryl acetate, and saccharide isomerate. This product for topical use belongs to OTC drugs, which are not reviewed and approved by the FDA. Another example is β-carotene, which is one of the ingredients in supplements for pregnant women (PregVit^®^) recommended for vitamin and mineral supplementation (2 μg of additional β-carotene corresponds to 1 μg of retinol). Β-carotene is also administered as an adjunct to skin diseases related to hypersensitivity to sunlight (erythropoietic protoporphyria) or pigmented disorders (e.g., vitiligo) [381].

Carotenoids accumulated in the skin may be a marker of their bioavailability. Traditionally, measurement methods require an invasive approach and taking into account metabolites resulting from metabolic changes, e.g., biopsy of adipose tissue or measurement of carotenoid concentrations in blood serum [382]. The determination method is HPLC with detection of UV/Vis or mass spectrometry, which is recommended for the analysis of β-carotene [383]. Currently, non-invasive methods are available, e.g., via reflectance spectroscopy (RS), Raman spectroscopy, or heteroflicker photometry to measure macular pigment optical density. Further development of measurement methods is expected that will allow their use in medical offices, especially since the usefulness of the carotenoid status in the skin has been demonstrated not only as a marker of consumption and accumulation of antioxidant status but also as one of the biomarkers of anti-aging [384].

It Is expected that the use of carotenoids will increase in the future due to the demand from the food and pharmaceutical industries. There is therefore a need to develop new metabolic engineering techniques to provide a source of efficient plant raw materials. Examples include crocin and apocarotenoids, which are responsible for yellow, orange, and red colors. The main source of these pigments is precious saffron; therefore, genetic engineering methods are used to increase their accumulation in plants [385]. However, apocarotenoids are beyond the scope of this review.

To increase the stability and bioavailability of carotenoids for various applications, such as food fortification, cosmetic products, and pharmaceutical preparations, nanotechnology is useful [386]. In the case of carotenoids, nanocapsulation based on polymers and lipids has proven to be the most useful so far. Various forms of nanoparticles, in addition to their advantages, have their limitations. For example, nanoemulsions require the addition of high concentrations of surfactants and co-surfactants for stabilization [387].

In the case of liposomes, it is difficult to obtain appropriate retardability and low encapsulation efficiency [388]. In turn, SLN limits the possibility of changing the physical state under the influence of temperature [389]. In the case of NLC, the detected cytotoxicity and surfactant activity are controversial [390]. The above limitations seem to be overcome by polymer nanoparticles, which are the greatest hope for efficient and effective encapsulation of carotenoids.

## 7. Materials and Methods

This narrative review is based on the Pubmed database. An open search of the PubMed database using the following terms: (carotenoids) OR (photoprotection) OR (skin) yielded 946,112 results from 1974–2023. To identify studies assessing the importance of carotenoids in relation to skin photodamage and photoprotection, the following keywords were used in the search: (skin) AND (photoprotection) AND (carotenoids). We limited the search to articles published in English between 1974 and 2023. A series of 152 articles were collected, of which 89 met the search criteria. The collected database included 65 review articles and 24 clinical studies, meta-analyses, and randomized clinical trials, of which only 14 met the inclusion criteria. Additionally, articles from the literature included in selected articles were added. Ultimately, 390 articles were included, which were sorted thematically and individually assessed by senior authors, specialists in dermatology and plastic surgery.

Chemical structure has been created using PubChem Sketcher V2.4 based on canonical SMILES, which is available online at the webside: https://pubchem.ncbi.nlm.nih.gov//edit3/index.html (accessed day 11 October 2023).

## 8. Conclusions

Intensive research on carotenoids dates back to the end of the 20th century. Their activity, mainly as scavengers of reactive oxygen species, has been confirmed in in vivo and in vitro studies. The subject of research of choice is research on skin well-being and protection against photodamage, as these compounds are natural light filters synthesized by many organisms. Their importance for health is currently undergoing a renaissance, as evidenced by new research devoted to improving the bioavailability and stability of these compounds and many health aspects, starting with the skin as a protective barrier and ending with mental health. A special issue dedicated to carotenoids by Scientific Reports has been announced for 2024.

## Figures and Tables

**Table 1:** Skin phototype classifications.

Classification	Criterion	Phototypes	Application	Ref.
Fitzpatrick (FSP), 1975	skin and eye color	FZ1–FZ6	burn risk, UVR tanning, skin cancer risk	[19]
Glogau Scale, 1994	photos	G1–G4	the degree of photoaging in Caucasians	[26]
Fanous Classification, 2002	race and genetics	1–6	laser resurfacing, chemical peels, and dermabrasion	[27]
Taylor Hyperpigmentation Scale, 2006	skin color	15 skin hues and up to 100 gradations of different colors of pigmentation	dyschromias	[28]
The Baumann Skin Type Solution, 2006	hydration, sensitivity, pigmentation, and elasticity of the skin	16 skin types	a guide to proper skin care techniques for various complexions	[29]
Goldman World Classification of Skin Types, 2002	skin color, race, and ethnicity	Indian, Asian, Arabian, Mediterranean, Hispanic, African, European, and Caucasian	risk of burning, tanning, and postinflammatory hyperpigmentation	[30]
Skin Classification System, Satoh and Kawada 1986	Japanese skin type: JST	JST I–III	assessment of skin sensitivity to UVR, sunburn, and tanning	[31]
Lancer Ethnicity Scale (LES), Lancer 1998	geographical location and heredity	LES I–V	predicting treatment effectiveness for patients undergoing laser surgery or chemical peels	[32]
Modified Fitzpatrick skin type, 2018	characterization of skin types in Indian individuals	I–VI	to assess phototype, skin color, and the ability to burn or tan	[33]
Roberts Skin Type Classification System, 2006	the skin’s likely response to insult, injury, and inflammation	FZ (types 1–6) H (types 0–6) G (types 1–5) S (types 0–5)	phototype, hyperpigmentation, photoaging, and scarring	[34]
Roberts Scarring (S) Scale, 2006	scar morphology, a complete skin examination	S0 Atrophy, S1 None, S2 Macule, and S3 Plaque within scar boundaries S4 keloid, S5 keloidal nodule	the determination of the short- and long-term effects of numerous medical treatments	[34]
Roberts Hyperpigmentation (H) Scale, 2006	the history of postinflammatory pigmentation	H0 Hypopigmentation H1: Minimal and transient (1 year) hyperpigmentation H3: Moderate and transient (1 year) hyperpigmentation H5 Severe and transient (1 year) hyperpigmentation	the evaluation of the propensity for pigmentation	[34]
Von Luschan’s chromatic scale (VLS), 1897	36 opaque glass tiles compared to the patient’s skin	1–36	the classification of race by skin color	[35,36]
Willis and Earles Scale, 2005	skin color, reaction to UV radiation and pigmentation disorders, injury, and inflammation	The lighter skin, the higher the risk of sunburn. The darker skin, the higher the risk of hyperpigmentation.	the classification of the skin color of people of African descent; it anticipates the risk of pigmentary disorders and the sun’s response	[37]
The Griffiths Photonumeric Scale, 1992	degrees of photodamage	a 9-point visual scale that uses photographs	the classification of the photoaged skin through the severity of cutaneous photodamage	[38]
The Score of Intrinsic and Extrinsic Aging—SCINEXA, 2009	intrinsic aging (uneven pigmentation, fine wrinkles, lax appearance, reduced fat tissue, and the presence of benign skin tumors). extrinsic aging (yellowness, coarse wrinkles, elastosis, telangiectasias, and the presence of malignant skin tumors)	5 items indicative of intrinsic skin aging and 18 items characteristic of extrinsic skin aging	the evaluation of intrinsic and extrinsic skin aging	[39]
The Lactic Acid Stinging Test (LAST), 390. Frosch and Kligman, 1977	the intensity of the stinging immediately after the application of the lactic acid solution, after 2.5 min and 5 min	a 4-point scale (0 = none, 1 = mild, 2 = moderate, 3 = strong)	the diagnosis of sensitive skin	[40,41]
The confocal CSIESA (confocal score for the assessment of intrinsic and extrinsic skin aging) score, 2022	reflectance, confocal microscopy, and imaging	epidermal disarrangement score (range 0–12), epidermal hyperplasia score (range 0–9), dermal score (range 0–15)	the assessment of intrinsic and extrinsic skin aging	[42]
skin cancer phototype (SCP) classification, 2018	tendency to burn (4 answers) and ability to tan (4 answers)	1–4 SCP classes	skin cancer risk	[43]
The Eumelanin Human Skin Color Scale, 2022	eumelanin-generated using diffuse reflectance spectrophotometry (DRS)	eumelanin low (EML), <25; eumelanin intermediate low (EMIL), 25 to <50; eumelanin intermediate (EMI), 50 to <75; eumelanin intermediate high (EMIH), 75 to <100; and eumelanin high (EH), ≥100	describe human constitutive skin color, prediction of skin cancer risk	[44]
Del Bino °ITA color categories, 2013	the individual typology angle (°ITA) based on colorimetric parameters	°ITA > 55-very light; 41 < °ITA < 55-light; 28 < °ITA < 41-intermediate; 10 < °ITA < 28-tan; −30 < °ITA < 10-brown; ITA < −30-dark	the degree of constitutive pigmentation of the skin, ultraviolet radiation (UVR) sensitivity	[45]
An objective skin-type classification system, Seo 2022	measurements using Tewameter^®^, pH-meter^®^, Corneometer^®^, Sebumeter^®^, Cutometer^®^, Spectrophotometer^®^, PRIMOS^®^ Lite, and Janus^®^	five main categories (sensitivity, hydration, oiliness, elasticity, and skin tone); five corresponding subcategories (erythema, roughness, pores, wrinkles, and pigmentation, respectively)	reference values applicable to the Korean population	[46]

**Table 2:** The effects of UV, VIS, and IR radiation on human skin [57,58,59,60,61,62,63].

Subdivision of the Main Ranges	Penetration Depth	Mechanism of Action	Unfavorable Acute and Chronic Exposure Symptoms	Favorable Skin Symptoms
Infrared radiation (IR); Wavelength 10^6^–780 nm; Frequency 3 × 10^11^–3.8 × 10^14^ Hz				
near IR (NIR) (IRA) (760–1400 nm)	subcutaneous tissue, the dermis	ROS formation, protease expression, increasing mast cells, ECM degradation, apoptosis-related protein expression (AP-1), tryptase expression, heat shock proteins, MMP-3 and MMP-13, MMP-1, proteins; decreased: synthesis of procollagen I	Heat stress, ab igne erythema, characterized by lesions of the pigmented reticular skin with telangiectasias, thermal pain and circulatory collapse, photoaging, squamous cell carcinoma or Merkel cell carcinoma, and premature skin aging	photoprevention of the skin against UV, treatments for wrinkles and sagging, stimulating wound healing, promoting hair growth, alleviating pain and inflammation, LLLT, and PBM
mid-IR (IRB) (1400–3000 nm)	the upper epidermis	-	felt in the form of heat	-
IRC (3000 nm–1 mm)	stratum corneum	-	felt in the form of heat	-
Visible light (VL); Wavelength 780–400 nm; Frequency 3.8 × 10^14^ × 10^14^–7.5 × 10^14^ Hz				
Blue Light (400–500 nm); Yellow Light (560–590 nm); Red Light (620–750 nm)	penetrates skin from the epidermis into the dermis more deeply from blue to red light	ROS generation (400–450 nm); alterations in fibroblast morphology and ECM	induction pigmentation in high doses, generation of a heat sensation, induction erythema	photorejuvenation treatment of atopic dermatitis, eczema, and antimicrobial PDT by exposure to low-power visible light emitted by neon, diode, and argon lasers
Ultraviolet radiation (UV); Wavelength 400–100 nm; Frequency 7.5 × 10^14^–3 × 10^15^ Hz				
UVA (400–315 nm)	70–80% epidermis, 20–30% deep dermis	ROS generation, degeneration of collagen and elastin, fibroblast, vessel, and connective tissue damage, thickening of the stratum corneum, epidermal hyperplasia, dermal inflammation, the synthesis of MMP, slow fibroblast proliferative mutations in mtDNA in fibroblasts, a depletion of Langerhans cells, higher numbers of infiltrating leucocytes, and mast cells	IPD, PPD, irregular post-inflammatory pigmentation, sun spots, inflammation, suppression of the immune system, skin aging: decreased wound healing	
UVB (315–280 nm)	70% of the stratum corneum, 20% of the viable epidermis, and 10% of the upper part of the dermis	Keratinocyte, melanocyte, Langerhans cell damage, degradation of collagen and elastin, induces MMP-1 and MMP-2, ROS, abundant pro-inflammatory IL-1-family proteins, IL-1α, IL-1β, IL-18, and IL-33, infiltration of inflammatory leukocytes, induction of immunosuppression, DNA repair, or apoptosis	the neo-melanization (delayed pigmentation), inflammation, skin erythema, the skin photoaging; actinic keratoses, leukoplakia, malignant skin cancer; SCC; BCC;	Vitamin D synthesis, protection, and uniform pigmentation
UVC (280–100 nm)	absorbed in the outer dead layers of the epidermis	DNA damage	erythema, thinned epidermis, carcinogenic	UVC is absorbed by the stratospheric ozone layer

Abbreviations: immediate pigment darkening (IPD); inducing persistent pigment darkening (PPD); squamous cell carcinoma (SCC); basal cell carcinoma (BCC); matrix metalloproteinases (MMP), reactive oxygen species (ROS), low-level light therapy (LLLT), photobiomodulation (PBM), activator protein (AP), extracellular matrix (ECM) components, mitochondrial DNA (mtDNA), and photodynamic therapy (PDT).

**Table 3:** Clinical trials concerning application of carotenoids for skin photoprotection.

The Aim of Therapy	Composition of Supplement	Study Type	Population; Dose; Experiment Time	The Examined Parameters	Ref
PLE	Lycopene, β-carotene, and *Lactobacillus johnsonii*	a randomized, placebo-controlled, double-blinded study	*n* = 60; daily doses of 100 J/cm^2^ UVA1; 12 weeks	expression of ICAM-1 mRNA	[121]
Photoprotection	Lycopene, β-carotene, α-tocopherol, and selenium	a clinical trial	*n* = 25, two tablets per day; sun-simulated UVR (SSR); 7 weeks	skin color, minimal erythemal dose, sunburn cells (SBCs), p53, pigmentation index, and levels of lipoperoxides	[122]
Reduction of oxidative stress markers and erythema in human skin exposed to UVR	vitamin E and β-carotene	a clinical trial	*n* = 16; α-tocopherol (n = 8; 400 IU/d) or β-carotene (n = 8; 15 mg/d); 120 mJ/cm^2^ UVR; 8 weeks	the skin malondialdehyde, total glutathione	[123]
Reduction of facial wrinkles and erythema	β-carotene, Ataulfo mango	a randomized clinical pilot study	*n* = 36; 85 g or 250 g of Ataulfo mango; four times per week; 16 weeks	skin carotenoids, the wrinkle length (L) and width (W), and a severity (S) score	[124]
Reduction of immediate erythema induced by UVB	lycopene in capsule and tomato paste	an interventional, randomized, comparative study	*n* = 20; UVB wavelength emitted by UV radiation simulator; 10 weeks	serum lycopene, minimal erythematous dose, maximum erythema	[125]
Protection from broadband UVB-induced threshold erythema formation	7.5 mg lycopene, 2.9 mg phytoene and phytofluene, 0.4 mg β-carotene, 2.8 mg tocopherols from tomato extract, and 2 mg carnosic acid from rosemary extract per capsule	a double-blind, randomized, placebo-controlled study	*n* = 149; 1.25 MED of UVB; 2 soft gel capsules per day; 12-weeks	MED, the intensity of erythema formation; IL6 and TNFα, carotenoid plasma levels	[126]
Protection against UVR-induced erythema	synthetic lycopene, a tomato extract (Lyc-o-Mato), and a drink containing solubilized Lyc-o-Mato (Lyc-o-Guard-Drink).	a randomized clinical trial	*n* = 36; lycopene (about 10 mg/day); a solar light simulator; 12 weeks	Lycopene, phytofluene, and phytoene serum levels, total skin carotenoids, MED, an index of erythema intensity, and reddening of the skin	[127]
Photodermatoses, pigmentation disorders	β-carotene (25 mg) and canthaxanthin (35 mg) per pill	a clinical trial	*n* = 185, 4–6 pills in photodermatoses and 4 pills in pigmentation disorders	the erythematous response, skin color	[128]
UVA-induced skin pigmentation	Nutrilite™ Multi-Carotene Supplement	a double-blind, placebo-controlled, randomized clinical trial	*n* = 60 (Fitzpatrick types II–IV); 12 weeks	MED, MPPD, and skin carotenoid levels	[117]
UVB-induced erythema	Seresis (β-carotene and lycopene), vitamins C and E, selenium, and proanthocyanidins	a clinical, randomized, double-blind, parallel-group, placebo-controlled study	*n* = 8, the pilot study, 2 capsules per day for 16 weeks; *n* = 48, the main study, 3 times per day for 12 weeks	MMP-1, MMP-9	[129]
Skin photoprotection, physiological parameters	Lutein and zeaxanthin; 20% dispersion of lutein in safflower oil; 5% lutein dispersed in butylene glycol	a randomized controlled trial, a double-blind, placebo-controlled study	*n* = 40; orally, topically, or in combination (both oral and topical routes); lutein 50 ppm/zeaxanthin 3 ppm; 2× per day; 12 weeks	superficial lipids, skin hydration, photoprotective activity, skin elasticity, and skin lipid peroxidation—malondialdehyde	[130]
Skin and eye function	(3 mg lutein, 45 mg l-ascorbic acid, 5 mg dl-α tocopherol, and 2.5 mg α-lipoic acid) B (13 mg carotenoid, 2 mg lycopene, 30 mg dl-α-tocopherol, 60 mg l-ascorbic acid, and 10 mg polyphenol)	a randomized, double-blind study	*n* = 50 smokers; the midday sun for 2 h a day; 2 capsules per day; 8 weeks	surface lipids, skin hydration, and oxidative stress on the blood serum	[131]
Photoaging	β-carotene	a randomized controlled trial	*n* = 30; 30 and 90 mg/day; 90 days	the type I procollagen, MMP-1, fibrillin-1 mRNA, UV-induced thymine dimer, and 8-hydroxy-2′-deoxyguanosine formation	[132]
Tolerance to UVR exposure	1400 mg of EPA + DHA, 120 mg of GLA, 5 mg of lutein, 2.5 mg of zeaxanthin, and 1000 IU of vitamin D3	a clinical trial	*n* = 28; 4 capsules per day; 8 weeks	MED	[133]

Abbreviations: Prevention of polymorphic light eruption (PLE); minimal erythemal dose (MED); UVA-induced minimal persistent pigmentation dose (MPPD); matrix metalloproteinases (MMP); eicosapentaenoic acid (EPA); docosahexaenoic acid (DHA); gamma-linolenic acid (GLA).

**Table 4:** Examples of nanoformulations containing carotenoids.

Type of Compound—Loaded	Type of Nanoformulation	Preparation Method	Kind of Delivery/Aim of Study	Application	Ref.
AST	egg phosphatidylcholine liposomes	a lipid hydration method	topical, iontophoretic transdermal delivery	UV-induced skin damage	[315]
β-carotene	PCL nanofibers	the electrospinning technique	topical delivery	anti-inflammatory effects	[316]
AST	NE in the presence of Cremophor^®^ EL and Labrafil^®^ M 1944 CS functionalized with CMCS	a convenient low-energy emulsion phase inversion method	dermal and transdermal delivery	drug delivery	[317]
AST	hydrogels/lipogels	Carbopol 974P, lecithin, isopropyl myristate, propylene glycol, and ethanol	topical	anti-aging	[318]
AST	liposomes	a high-pressure homogenizer (phosphatidylcholine from soy, ethanol)	topical	anti-inflammatory effect on AD	[319]
AST	NLC	melt emulsification-ultrasonic technique	topical	the development of cosmeceutical	[320]
lycopene	NLC	high-pressure homogenization	topical	the effect of surfactant on stability	[321]
lycopene	NLC, SLN	a combination of high-shear homogenization and ultrasound methods	orange drink as a model food system	food fortification	[322]
lycopene	NE	high-pressure homogenization with food-grade biopolymeric OSA-modified starch as the emulsifier	simulated gastrointestinal model	the design of lycopene-fortified delivery systems	[323,324]
β-carotene	NLC	the high-pressure homogenization of the preemulsion	functional ingredient in beverages	lipidic colloids in food systems	[325]
carotenoid extract of *Ipomoea batatas* L.	NE	mixing an appropriate proportion of carotenoid extract, Tween 80, PEG 400, soybean oil, and deionized water	gastric and intestinal condition model	inhibiting tumor growth in mice	[326]
carotenoids from paprika, oleoresin	NE	whey protein, gum Arabic, and soy lecithin through a high-pressure homogenizer	the physical and chemical stability during storage	designing delivery systems to encapsulate and stabilize lipophilic molecules	[327]
paprikaoleoresin, and carotenoids	NE	a high-speed homogenization and high-power sonication of the paprika oleoresin dispersed in aqueous Tween 40	orally administered to Wistar rats	in vitro and in vivo antioxidant properties, the effects of nonionic surfactants on liver	[328]
β-carotene	SLN	homogenization-evaporation method of SC, WPI, or SPI	in vitro	stability, cytotoxicity, and cellular uptake by Caco-2 cells	[329]
β-carotene	SLN	the hot, high-pressure homogenization method	the physical and chemical stability of SLN	impact of surfactant properties on SLN and β-Carotene degradation during storage	[330]
β-carotene, lycopene, β-cryptoxanthin, lutein from courgette (zucchini), red pepper, and tomato	micelles	the micellarization	in vitro digestion procedure	carotenoid bioavailability	[331]
β-carotene	NLC	high-pressure melt-emulsification	Physical stability	functional ingredient in beverages	[325]
carotenoids	nano-sized hydrosols	polymer- and surfactant-controlled precipitation and phase separation techniques	Preparation and characterization	the pigmentation of foods and pharmaceutical preparations	[332]
carotenoids extracted from halophilic Archaea	O/W micro, and NE	high-pressure homogenization and a spontaneously formed microemulsion	the structural characterization and stability	functional food applications	[333]
vitamin C and β-carotene	liposomes	co-encapsulated liposomes using hydrophilic and hydrophobic cavities by the ethanol injection method.	in vitro gastrointestinal digestion	Storage stability, antioxidant activity	[334]
lycopene	transfersomes, ethosomes	the method of Simoes et al. [335]	dermal delivery	formulations for antioxidant treatment	[311]
AST	NLC	melt emulsification–sonication technique	optimizing NLC composition (Tween 80 and lecithin as emulsifiers, and oleic acid and glyceryl behenate as lipids)	designing an AST-loaded NLC	[336]
AST	biopolymeric nanosphere	Chitosan and salmon sperm DNA were used to fabricate a water-dispersible nanocarrier system	in vitro study of the cellular uptake and antioxidant capability	absorption through endocytosis by intestinal epithelial cells	[337]
β-carotene	liposome	the hydration of proliposomes (phospholipid) obtained by spray drying using ultra-agitation	physico-chemical stability and feasibility in yogurt	production of fortified food	[338]
β-carotene	NE	a two-stage high-pressure homogenization	the physicochemical properties	optimization of the conditions for preparing NE	[339]
β-carotene	NLC	the solvent diffusion method	for use in foods and oral administration	Optimization of the formulation	[340]
β-carotene	SLN/NE	homogenizing lipid (cocoa butter and/or hydrogenated palm oil), surfactant (Tween 80), and water (≈60 °C), and then cooling	the physical and chemical stability	particle aggregation and β-carotene degradation	[341]
β-carotene, α-carotene, lutein	polymeric nanocapsule	the interfacial deposition of the preformed polymer	The encapsulation efficiency and the β-carotene retention	To produce, characterize, and evaluate the stability	[342]
β-carotene	polymeric nanosphere	the water-in-oil solvent displacement method	Encapsulation of β-carotene in PLA	protection against oxidation	[343]
lycopene	NE	an emulsification-evaporation method	antioxidant activity of lycopene-enriched tomato extract	production of fortified food	[344]
lycopene	NLC	high pressure homogenization	topical administration	the stability and degradation of lycopene	[345]
lycopene	lipid-core nanocapsules	the interfacial deposition of PCL	physicochemical characterization	stability study	[346]
lutein	NE	A Microfluidizer^®^ Processor	a beverage fortification	bioavailability study	[347]
lutein	NLC, NE, SLN	high pressure homogenization	dermal delivery	protect skin from photo damage	[348]
lutein	polymeric nanosphere	CS/γ-PGA nanoencapsulation	the solubility of lutein	optimization of the formulation	[349]
lutein	polymeric nanosphere	solvent displacement method	the particle size and particle size distribution (PSD)	The effects of processing parameters, emulsifiers, and stabilizing mechanisms	[350]

Abbreviations: atopic dermatitis (AD); nanostructured lipid carriers (NLC); solid lipid nanoparticles (SLN); Octenyl succinate anhydride (OSA); food-grade sodium caseinate (SC); whey protein isolate (WPI); soy protein isolate (SPI); oil-in-water (O/W); nano-emulsions (NE); polylactic acid (PLA); poly(ε-caprolactone) (PCL); chitosan (CS); poly-γ-glutamic acid (γ-PGA); carboxymethyl chitosan (CMCS).

## Data Availability

Not applicable.
